# Quality of Life after Spinal Cord Injury: A Multiple Case Study Examination of Elite Athletes

**DOI:** 10.3390/ijerph17207437

**Published:** 2020-10-13

**Authors:** Agata Goraczko, Grzegorz Zurek, Maciej Lachowicz, Katarzyna Kujawa, Wiesław Blach, Alina Zurek

**Affiliations:** 1 Clinic of Neurorehabilitation, 54-519 Wroclaw, Poland; agagoraczko@gmail.com (A.G.); katarzyna.kujawa@awf.wroc.pl (K.K.); 2 Department of Biostructure, University School of Physical Education, 51-612 Wroclaw, Poland; maciej.lach93@gmail.com; 3 Department of Sports Didactics, University School of Physical Education, 51-612 Wrocław, Poland; wieslaw.blach@awf.wroc.pl; 4 Institute of Psychology, University of Wroclaw, 50-137 Wrocław, Poland; alina.zurek@uwr.edu.pl

**Keywords:** spinal cord injury, quality of life, elite athletes

## Abstract

A three-times World Champion in BMX (an acronym for Bicycle Motocross) dirt jumps, a Junior World Champion in ski jumping, and a European karate Champion sustained spinal cord injuries at the cervical and thoracic level. Such a severe trauma is tantamount to the end of a professional sporting career. In such a situation, the athlete’s life significantly changes in every aspect of it: health, professional, and social. The greatest sports champions have not yet been portrayed in the context of a strategy they used to deal with an abrupt end of a professional career due to severe injury. A semi-structured interview was conducted with study participants who additionally filled out the WHO Quality of Life Scale. This multiple case series presents the quality of life in elite athletes as well as the social activities they have undertaken regardless of the tragic accident. The results of the research indicate that these people are characterized rather by a positive sense of quality in life, and the way they function in a difficult situation is an inspiration to others.

## 1. Introduction

Spinal cord injury (SCI) is a serious condition usually associated with irreversible loss of motor functions, deterioration of the financial situation, social isolation, and psychological problems [1,2]. The majority of people sustaining such injury are no longer able to work, becoming dependent on their family and health care support, all contributing to a significantly impaired quality of life (QoL) [3]. The World Health Organization defines the quality of life as individuals’ perception of their position in life in the context of the culture and value systems in which they live and in relation to their goals, expectations, standards, and concerns. It is a broad-ranging concept affected in a complex way by the person’s physical health, psychological state, level of independence, social relationships, personal beliefs, and their relationship to the salient features of their environment [4,5]. It has been found that quality of life is deteriorating as a result of SCI and may be affected by personal (sociodemographic and psychological), cultural (race and ethnicity), economic, and environmental (availability of medical care, quality of education, employment opportunities, and place of residence) factors [6]. People with SCI on average tend to report a decreased feeling of well-being and grade their physical, mental, and social health lower than nondisabled persons [7].

Frequent problems in people after SCI include chronic pain and spasticity, with severe pain being particularly associated with a lowered quality of life [8,9,10]. Moreover, breaking the continuity of the spinal cord results in many disorders related to the basic functions of the urinary, digestive, respiratory, and cardiovascular systems, and sexual activity [11,12,13]. The quality of life of people following high spinal cord injury is relatively poorly described in the literature [14,15].

For physically active people, who consider sport their passion, functional disability takes an even greater dimension of tragedy. The injured person loses what was “their whole life” and faces a tremendous challenge to find new meaning and motivation in life [6]. Former elite athletes, with significant achievements to their credit, for whom sporting activities also had a professional dimension, represent a separate, special group of people with disabilities [16]. Practicing sport, especially extreme sport, involves an increased risk of serious injuries, including SCI [6]. Despite the recognition of sports as a significant contributor to the etiology of SCI, only several studies appear to have explored the epidemiology of SCI caused by sports [17]. In the USA, approximately 8.7% of new cases of such injuries are related to sporting activities. The greatest risk of cord injuries is found in soccer, ice hockey, wrestling, skiing, rugby, and snowboarding [17,18]. Research shows that in skiing and snowboarding the frequency of SCI is 0.01 and 0.04 per 1000 days of practice respectively [16]. American football, which is a contact sport, is also treated as a high-risk sport in the context of cervical cord injuries with potential neurological deficits [19]. A study by Ackery et al. (2007) shows that the number of spinal cord injuries has been increasing in recent years [20]. This may be due to the desire to perform more and more extreme stunts and the increase in competition in sport. 

Recent literature suggests that extreme sports experiences are often extraordinary, transcending, transforming and the opportunity to transcend the everyday experience may provide more motivation and inspiration than experiencing short-term thrills through risk-taking [21]. Filbay et al. (2019) found that former athletes had a similar physical component score and a better mental component score, compared to the norms of the general population. On the other hand, former athletes with physical impairment reported better mental health than the rest of the population [22]. The authors as well as Smith and MacManus (2009) pointed out factors that may associate with worse QoL: involuntary retirement from sport (injury or deselection), increased Body Mass Index and osteoarthritis, or musculoskeletal issues, a strong, exclusive athletic identity, and lack of pre-retirement planning and support services [22,23]. Moreover, Smith pointed out that there are gaps in the scope of programs to address broader adaptation issues and greater emphasis should be directed towards the psychological, social, and physical transitions experienced by elite athletes prior to and after retirement to ensure a positive adaptation into post-sports life [23]. Lemenz (2015) found considerable support for superior longevity outcomes for elite athletes, particularly those in endurance and mixed sports [24]. On the other hand, process of athlete commodification (created by the consumption, media, fans, etc.) influences the quality of transition into lives after sport, seeing that very often they are not enough prepared for such a change [25].

Keer (2012) shows, that the range of motives for extreme sport participation included: goal achievement, risk-taking, social motivation, escape from boredom, pushing personal boundaries and overcoming fear, as well as connecting with the natural environment, and pleasurable kinaesthetic bodily sensations from moving in water or air [26]. In a sports competition, a successful athlete must have the psychological and mental characteristics as the appropriate level of self-motivation, self-awareness, and self-control needed to have achievements [27]. The findings of Sootmaker research (2018) suggest that BMX (an acronym for Bicycle Motocross) professionals are intrinsically motivated to take risks and set goals while placing little value on objective rewards [28]. Widyastuti and Dimyati found that karate fighters are characterized by fighting spirit, creativity, practical intelligence, control and self-control capacity, the spirit of sacrifice, intelligence, motivation, combat power, aggressiveness, tenacity, and quick thinking [27]. The results of Vegard’s study (2018) show that psychological factors that regulate emotional states, coping strategies, constructive mindset, and motivation may have important meaning for World Cup ski jumping performance [29].

The literature on practicing sport by people after SCI is well established [1]. Numerous studies are indicating multiple health, social and psychological benefits of being physically active by people with disabilities [30,31]. Ciampolini (2017) assessed the quality of life in Brazilian wheelchair tennis athletes and compared the perception scores between competitive and elite athletes. The elite group has been found to exhibit statistically higher perceptions in the physical domain and the total QoL score [1]. McVeigh et al. (2009) compared the quality of life and social integration of people with spinal cord injury involved in sport with those who are not involved in sport [30]. In this study, the results of the Community Integration Questionnaire (CIQ) and Reintegration to Normal Living Index RNL were higher among participants involved in sports activities (*p* < 0.05) and correlated positively with quality of life. People who took part in sports before spinal cord injury were more likely to be involved in sports after the injury.

However, to our knowledge, so far only two articles have been published that concern former athletes who have suffered from SCI as a result of sports [6,32]. The subject of a study by Smith and Sparkes (2005) was narratives of hope in the life of men who experienced SCI during rugby games. Most of the respondents felt a strong hope, which was based on their belief in recovery. In a study by Badenhorst (2018), which involved 90 individuals with rugby-related spinal cord injury in South Africa, they rated their quality of life higher than those in the control group [6].

This work aims to assess the quality of life and methods of coping with the spinal cord injury situation in three elite athletes after their sporting careers have ended due to a serious spinal cord injury.

The respondents agreed to participate in the project and to publish the results obtained. The plan of the project was positively reviewed by the Bioethics Committee of the University School of Physical Education in Wroclaw.

## 2. Materials and Methods

A question about participation in the study was sent to 23 athletes from different countries of the world. A positive response was obtained from 3 people, who gave an interview and returned a completed personal questionnaire and WHOQoL (The World Health Organization Quality of Life questionnaire) so they were included in the study. These were people from the United Kingdom, Austria, and Poland. The criteria for inclusion in the study required that participants had represented their country in sporting competitions and that they had won the championship title in a sports event at the international level. An additional criterion was a communicative knowledge of English or Polish. The consent of the Senate Research Ethics Committee of the University School of Physical Education in Wroclaw for conducting a research project was obtained.

The first participant (P1) had been training to jump on a BMX bike since he was three years old. He became a three-time gold medalist of the biggest BMX sporting events—the Gravity Games and the X-Games. During the World Cup, while performing his own double backflip figure, he lost his orientation in space and fell to the ground hitting his head against the ground. As a result of the accident, he sustained a spinal cord injury at the C3/4 (cervical) level and quadrupedal paralysis. He now moves in a wheelchair, which he controls using sensors placed in the headrest.

The second participant (P2) is a former ski jumper, double junior world champion. During one of the training sessions, during the flight, his foot slipped out of the ski boot and an uncontrolled landing and fall caused damage to the spinal cord at the C6/7 level. He moves in a wheelchair, which he operates using his upper limbs on his own. Thanks to intensive rehabilitation he can maintain an upright posture, take a few steps on his own, and cover a short distance supported with a pair of crutches.

The third participant (P3) is a former European karate champion. During a sports camp, while moving on a rope suspended at the height of the first floor, her hands slipped and she fell to the ground, causing paraplegia (her spinal cord is damaged at the TH11/12 (thoracic) level). She moves on her own in a wheelchair with the help of her upper limbs.

Participation in the study consisted of giving an interview via an Internet communicator and filling an online WHOQoL and personal questionnaires. The participants agreed to use the collected material for scientific publications. Before the interview participants read consent of participation, which included: title and purpose of the project, explanation of procedures, and confidentiality. At the beginning of the interview, the participants gave verbal consent to the conditions of the project. Sociodemographic data were collected by means of a personal questionnaire, which also included questions about the date of the accident, the level of spinal cord injury, the circumstances of the accident, and the greatest sporting achievements. The Numerical Rating Scale of Pain (which contains 11 degrees of pain severity between 0 and 10) was attached to the questionnaire.

Participants’ quality of life was examined using the shortened version of the WHO Quality of Life Questionnaire (version WHOQoL-BREF). Adaptations of the national WHOQoL scales were used. The scale contains two general questions and 24 questions that describe 4 domains: physical health, as well as social, psychological, and environmental aspects [33]. The WHOQoL BREF uses a five-level Likert scale, where athletes determine satisfaction (strongly agree, agree) or dissatisfaction (strongly disagree, disagree) or remain neutral by choosing the answer “neither agree nor disagree”. A higher score indicates a subjectively better quality of life. The WHOQoL scale is currently considered the most appropriate instrument for assessing the quality of life in people with traumatic spinal cord injuries [34,35].

All semi-structured interviews were conducted by the first author, who has many years of clinical experience working with people after SCI. Each of the interviews lasted about two hours, was recorded, and then transcribed. The thematic analysis of the interviews was conducted by the first author and then verified by competent jurors (the two subsequent authors). The thematic analysis consisted of the following stages: getting familiar with the data through several times open-minded reading; search for meanings and themes, organizing themes into a meaningful wholeness [36,37]. The content corresponding to four areas: physical, psychological, social, and environmental was chosen. It resulted in extending the quantitative data retrieved using the WHOQoL with the subjective world of experiences, feelings, and thoughts of the subjects.

The quantitative and qualitative research project conducted allowed the research team to reach the unique subjective world of experiences of SCI athletes.

## 3. Results

### 3.1. Information on the Respondents

Sociodemographic and health data of the participants is presented in Table 1.

### 3.2. Quality of Life—WHOQoL Results

To analyze the WHOQoL-BREF instrument results, the raw point values obtained for individual domains were recalculated on a scoring scale ranging 4–20, in line with the WHO recommendations [38]. The questionnaire is opened by two introductory questions on the quality of life rating and health satisfaction. For the first question, “How would you rate your QoL”, participants who answered “very poor”, “poor” or “neither poor or good” were classified as having a negative perception, while those who answered “good” or “very good” were classified as having a positive perception of their QoL. Analyzing the overall quality of life feeling, P1 perceived his QoL negatively, while the other two were positive. P1 described his general health as very poor, while P2 and P3 were classified as very good and good, respectively (Table 2). In the personal questionnaire, P1 indicated the occurrence of respiratory disturbances during sleep and severe pain. Furthermore, he was in bed at the time of the interview because of a pressure sore in the buttock area. These factors may have affected his perception of his health as very poor and may have affected his overall quality of life. P1 also indicated that he would rate his quality of life as very good and his health as good before the latter started to deteriorate. The highest quality of his life in each domain was perceived by P3.

### 3.3. Quality of Life—Analysis of the Interviews

The physical domain covers the following areas: pain, discomfort, energy and fatigue, mobility, sleep quality, daily activities, and work capacity. P1: “Right now I am isolated. I need to stay in bed all the time because of pressure sores. My health condition in the last 2 months has been terrible. I feel really bad”. P2 indicates that a very high pre-traumatic fitness level had a major impact on success in rehabilitation. He appreciates and considers every functional achievement to be a success, even though he is paralyzed as a result of the accident. The limitations also apply to aspects that are unseen for other people, such as excretion, eating, sweating. P3 indicates increased muscle tension in the lower extremities and spinal pains.

The psychological domain includes positive and negative feelings, spirituality, religion, thinking, personal beliefs concentration, body image, appearance, and self-esteem. The study participants cope with this area by finding new tasks and goals, and sources of motivation and strength. They also fill their life after the accident with new content and appreciate the value and importance of what they have. They said (P2 and P3): “How good it is to have an agile head and hands!”. In the context of psychological functioning, P2 clearly emphasized the sentence that he heard from his doctor informing him about the spinal injury: “I have to remind you that today you have a healthy head, a healthy mindset, and quite healthy hands, and these components ensure that you can lead quite a normal life’. Yeah and maybe this was the most important sentence in this whole journey which I’ve been making since that day.” A sentence uttered by P3 is also interesting: “The accident has taught me a lot. From the very beginning, I was positive and kept on fighting. I was cheerful from the beginning. Karate before the accident taught me perseverance and diligence, which later became very useful in this daily fight. A year after the accident I passed my driving license. Now I drive my own car. Less than a year after the accident I signed up for the Miss Polonia competition in a wheelchair. I would never have thought of that before”. P3 also said, “I’ve seen people look at me many times when I get out of the car and it’s a wonder that I’m in a wheelchair and can drive at the same time. People are also surprised how I take care of myself, that I look so good, that I do not lock myself among four walls but go out to people, I do a lot of things”. P3 attaches a lot of importance to how people react to her struggles, efforts, successes, and the inner strength that drives her to act. They build her sense of value and allow her to believe in herself.

The social domain contains relationships and social support. Interpersonal relations are very important for each of the participants: among family, friends, and in the professional context. Particular attention should be paid to P2’s and P3’s statements in the context of relations. P2: “You have to learn so many things and you always have a feeling that you aren’t ready for it, but it feels like somebody is pushing you into cold water. You always have to rely on other people because on your own it is impossible to do certain things. My family is my biggest support all the time, friends also, ski jumpers, my physiotherapist.” P3: “I’ve been half-orphaned since I was 8 years old, I was only raised by my dad. I also have two brothers who support me, and I can always count on them. I have great friends at the university who always help me with architectural barriers, and I don’t have any problems moving around either.”

The environmental domain covers the following areas of life: safety, security, physical environment quality, finances, information access, leisure activities, home environment, access. The living environment and recognition by others before and after an accident are crucial for the quality of life, wellbeing, and finding a permanent commitment to work. The sport continues to fill the lives of the participants, although no longer as an active athlete but as a trainer, advisor, coach, or motivator. It is heard in the words of P1: “I was very lucky because it was quite public. I was highlighted a lot from my crash. People in sports insurance were amazing: they have set up a fund and people donated money”. P2 formulated it as follows: “For me every message I get after my accident is so important because I’m not a robot, of course, I have my bad days, and on these bad days I try to remember what people wrote me. It’s a little exit door from this bad day, those messages I got are these exit doors, so I’m really happy to have these messages. Because they remind me that people had hope and because I can give some inspiration back to them. So, it is a circle, and to keep it circling it is important to go on, even if it is not pleasant on some days... I wanted to stay here, because of ski jumping and training. The connection with the ski jumping team is still here and it is always fun to watch them at their training. I have my friends here and an ex-girlfriend”. P3 is studying prosthetics and indicates that the dental institute is well adapted to people with disabilities. She is also active professionally, working in an active rehabilitation foundation as an instructor.

## 4. Discussion

In this article, an attempt has been made to illuminate the quality of life of former famous athletes, who finished their careers because of a sports injury. We have also tried to reflect on their social activities.

Analyzing the overall feeling of quality of life, P1 perceived his QoL negatively, while the other two respondents positively. P1’s score may be associated with poor health in the last four weeks, and the WHOQoL scale refers to this period of time. People with chronic tetraplegia experience more subjective sleep problems and worse quality of life than their able-bodied counterparts [39]. A study involving 270 individuals who sustained SCI found that poorer QoL was associated with secondary impairments (for example, neuropathic pain, urinary tract infection), activity limitations, and participation restrictions, but not with neurological level, completeness of injury, age, or time since injury [11]. Secondary impairments in the last four weeks have significantly influenced the WHOQoL score of P1.

In the Badenhorst study (2018) carried out among individuals with rugby-related spinal cord injuries, participants had higher QoL scores compared to other studies in non-sporting cohorts. This may result from analyzing the results of a specific group of subjects (former athletes), which is associated with initial better physical health and support by sports organizations [6]. This was also emphasized by P2 in his own research.

Qualitative research with people who have suffered an SCI through doing sports revealed that individuals with strong athletic identity before the SCI can have adaptation difficulties after their injury [40,41]. Smith (2005) found that most of the participants waited for the invention of a therapeutic method that will allow for a complete recovery. Medical unreliability is not taken into account, and the possibility of remaining disabled for the rest of one’s life is rejected [32]. On the other hand, athletic identity has also been reported as a factor able to promote recovery and is considered as a means to enhance long-term adjustment to disability [40]. In a Saban study (2015) conducted among wrestlers at different athletic levels, the national-level wrestlers achieved the highest score in the social domain. The author explains this result by pointing to the fact that national wrestlers are in touch with athletes from different countries through national and international competitions, which is how they meet new cultures, also making them active in social domains [42]. Similar findings were observed among athletes participating in the presented study, who were strongly involved in sports, social life, and motivational speech.

The findings reported by Ciampolini (2017) suggest that even though participation in high-performance sports may offer a stressful and exhausting environment, elite wheelchair tennis athletes from Brazil perceived themselves as having a higher QoL than competitive athletes [1]. In contrast, the results of a study by Yazicioglu (2012), involving 60 participants with physical disabilities, indicated that people participating in sporting activities appropriate to their degree of disability have a significantly higher quality of life and life satisfaction compared to people not involved in any sport [43,44]. Similar results were observed in the present research: even passive participation in competitions and training, and the involvement of a disabled former athlete in work for the benefit of the sport ensures the maintenance of a sports-related identity and is a source of both satisfaction and fulfillment. P1 is not involved in any sporting activity due to functional limitations. However, he has two children who regularly train BMX and is active in this environment, giving motivational speeches (for example for Liverpool players). P2 is in constant contact with the ski jumping community and prepares for the profession of a coach. P3 has started to train dancing in a wheelchair. Thus, the difficult life experiences of people who were forced to end their sporting career because of an accident can be a motivation for other athletes, for example, those experiencing professional burnout [45]. The life testimonies of the participants can be used to build a positive picture of life even after SCI.

## 5. Conclusions

Despite a traumatic accident, the sudden end of a successful sporting career, and a completely changed life, the respondents in this study positively described their quality of life. However, it is important to note the dependence of the result on the state of exacerbation or alleviation of physical symptoms associated with SCI. Each participant maintains a strong connection to the sporting environment and is socially involved. As celebrities, they are observed by their fans, which motivates them to act. The involvement of athletes after SCI in the sporting environment prevents them from losing their sporting identity and makes it easier to find themselves in a new life situation [42]. This may also serve as an example for other people with severe disabilities, encouraging them to engage in social activities. Former elite athletes with strong psychological characteristics may be a help for the athletes ending their sports careers, who have problems with adaptation into post-sports life.

Differences between respondents may stem from a variety of factors that have not been taken into account, such as different national health care systems and family support; these factors significantly affect the perception of the quality of life [2].

## Figures and Tables

**Table 1:** Sociodemographic and health data of study participants. P1—participant one, P2—participant two, P3—participant three, SCI—spinal cord injury, C—cervical, TH—thoracic.

Categorical Variables	P1	P2	P3
Age	40	28	23
Nationality	British	Austrian	Polish
Level of SCI	C3/4	C6/7	TH11/12
Age when injured	27	24	18
Marital status	divorced	single	informal relationship
Respiratory disorders during sleep	apnea, shallow breathing	none	none
Numerical Rating Scale	7	3	3

**Table 2:** WHOQoL (The World Health Organization Quality of Life questionnaire). Q1—Overall perception of the quality of life. Q2—Overall perception of health.

Domain	P1	P2	P3
Q1	3	4	4
Q2	1	5	4
Physical health	14	11	18
Psychological	13	15	19
Social relationships	13	12	20
Environment	16	16	17
